# Determining Physical Constraints in Transcriptional Initiation Complexes Using DNA Sequence Analysis

**DOI:** 10.1371/journal.pone.0001199

**Published:** 2007-11-21

**Authors:** Ryan K. Shultzaberger, Derek Y. Chiang, Alan M. Moses, Michael B. Eisen

**Affiliations:** 1 Department of Molecular and Cell Biology, University of California, Berkeley, United States of America; 2 Graduate Group in Biophysics, University of California, Berkeley, United States of America; 3 Department of Genome Sciences, Genomic Division, Ernest Orlando Lawrence Berkeley National Lab, Berkeley, California, United States of America; Tufts University, United States of America

## Abstract

Eukaryotic gene expression is often under the control of cooperatively acting transcription factors whose binding is limited by structural constraints. By determining these structural constraints, we can understand the “rules” that define functional cooperativity. Conversely, by understanding the rules of binding, we can infer structural characteristics. We have developed an information theory based method for approximating the physical limitations of cooperative interactions by comparing sequence analysis to microarray expression data. When applied to the coordinated binding of the sulfur amino acid regulatory protein Met4 by Cbf1 and Met31, we were able to create a combinatorial model that can correctly identify Met4 regulated genes. Interestingly, we found that the major determinant of Met4 regulation was the sum of the strength of the Cbf1 and Met31 binding sites and that the energetic costs associated with spacing appeared to be minimal.

## Introduction

The regulation of transcriptional initiation from individual eukaryotic promoters is often controlled by multiple cooperatively interacting transcription factors. These factors bind to separate sites in cis-regulatory sequences and physically interact with each other, either directly or through additional proteins, to activate or repress transcription [1], [2], [3]. These physical interactions among transcription factors must constrain how their binding sites can be positioned relative to each other and to the relevant promoters. Yet, there is often considerable variability in the order, orientation and spacing of binding sites for interacting transcription factors [4], [5], [6]. Understanding how the arrangement of sites is related to the stability of these complexes and their regulatory activity is essential if we are to understand the regulatory content of eukaryotic genomes.

To successfully model the binding of multi-meric complexes to different target sequences, many energetic contributions need to be considered. The affinity of each transcription factor for DNA varies considerably with the precise bound sequence, even among known in vivo targets [7], [8]. The stability of the entire complex is also dependent on how compatible the positioning of the sites are with the protein-protein interactions necessary to form the complex. Poorly positioned sites presumably introduce clashes or strain into either the complex or DNA which will, in turn, reduce the stability of the complex.

Here, we combine DNA sequence analysis and genome-wide expression data to discern the constraints on the arrangement of binding sites for transcription factors involved in regulating the synthesis of sulfur-containing amino acids in the yeast *Saccharomyces cerevisiae*. This work builds on our previous modeling of bipartite prokaryotic ribosome and σ^70^ binding sites [9], [10]. In both of these cases, initiation requires the cooperative binding of two independent components separated by a variable spacer, the Shine-Dalgarno and P site for ribosome binding sites, and the −10 and −35 for σ^70^ binding sites [11], [12], [13], [14]. Since there were a large number of characterized sites for these systems, we constructed a robust distribution of the allowable spacings between binding components. Assuming that the spacing that would induce the least amount of strain in the protein or in the bound DNA upon binding would be the most commonly observed, and that the frequency of occurrence of all other spacings would be directly related to the energetic consequence of using that spacing, we could model the energetic contribution of different spacings to the formation of a stable initiation complex.

Cooperatively acting transcription factors in eukaryotes are similar to the prokaryotic ribosome and σ^70^ in that they have independent binding components separated by variable spacers, but they are different in that the components are not physically linked upon binding and therefore can bind in different orders, orientations, and with greater variability in their spacing. We have devised a method to determine these additional physical constraints by optimizing an information theory based model against microarray data. We can use these optimized constraints to not only infer structural characteristics of the regulatory complex, but also to quantify the binding of these multi-meric complexes to different DNA sequences, and to accurately predict target genes.

Met4 is the major transcriptional activator of sulfur utilization genes in *Saccharomyces cerevisiae* even though it does not bind directly to DNA [15], [5]. Met4 stabilization is dependent upon at least two additional proteins. One of these is the centromere-binding factor (Cbf1) [15], whose DNA binding activity is stimulated by association with Met28 [16]. It has been suggested that the Cbf1-Met28-Met4 complex may be sufficient for activation of some genes, but coordination by a second factor is necessary for others [4]. We are interested in describing this coordinated system. The second stabilizing factor that we will study is Met31, a factor unique to sulfur regulation [17].

Neither the distance between Cbf1 and Met31 in functional Met4 stabilizing complexes, nor the distance between Met4 and the initiating polymerase is fixed [5]. We extended the information theory-based method we used to study prokaryotic translational and transcriptional initiation to model Cbf1 and Met31 interactions, allowing for the greater flexibility present in this system.

## Materials and Methods

### Cbf1 and Met31 binding models

**Figure 1:**
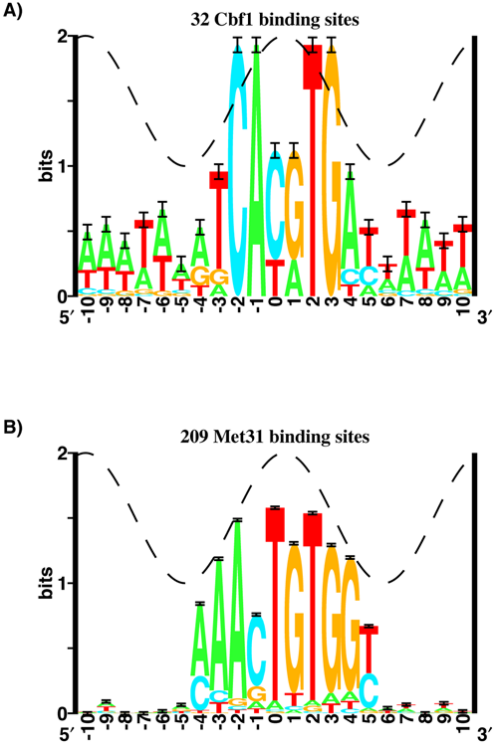
Cbf1 and Met31 sequence logos. Sequence logos were made as described in Materials and Methods. The height of each letter is proportional to the frequency of that base at that position. The height of the letter stack is the information content at that position. The cosine wave represents the helical twist of B-form DNA. The sequence logos were generated using the standard Delila programs [19], [21].

We built a weight matrix describing the sequence preferences of Cbf1 from 16 Cbf1 binding sites characterized by Wieland *et al.* [18]. Binding matrices were built using the standard **Delila** programs [19], [20]. Since Cbf1 binds as a homodimer, we used each sequence and its complement to build our model [21] (Fig. 1A). Because of the lack of experimentally verified binding sites for Met31, we modeled its binding by analyzing 21 non-divergently transcribed genes identified in a Met4 chromatin immuno-precipitation assay [3] (we selected genes with p<0.001). We used MEME [22] with the -tcm model and required at least 10 copies of a motif to identify sequences enriched in these target genes, from which we computed an initial Met31 weight matrix. We then scanned the entire genome for sites with greater than 10 bits of information against this model, identifying 209 sites, from which we constructed the Met31 weight matrix used in our analysis (Fig. 1B).

### Searching algorithm

Multi-component binding systems with variable spacing between components have previously been modeled [9], [10]. In the case of the prokaryotic ribosome and σ^70^, the binding components are physically connected. In both instances, deviations in the optimal spacing between components introduces strain in the bound complex and affects the binding energy [11], [12], [13], [14]. To model these multi-meric binders the following equation was used:
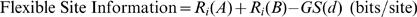
(1)where *R_i_*(*A*) is the relative strength, or individual information, of binding factor A, and *R_i_*(*B*) is the relative strength of binding factor B according to [20]. *GS*(*d*) is the gap surprisal (based on Tribus' surprisal function [23]), or penalty of having a spacing of *d* between sites A and B as determined by [9], [10]:
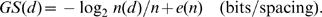
(2)
*n*(*d*) is the number of occurrences at spacing *d* and *n* is the number of total occurrences over the allowed values of *d*. *e*(*n*) is a small sample correction value [24], [9]. For our initial analysis of Cbf1 and Met31, we used a flat spacing distribution where all spacings have the same gap surprisal value of *GS*(*d*) = -log_2_(1/(*d_max_*–*d_min_*+1)), where *d_min_* is the shortest spacing between Met31 and Cbf1, and *d_max_* is the longest spacing. The distance between Met31 and Cbf1 is calculated between the zero positions of the binding components as with previous flexible models.

For the ribosome and the polymerase, the binding components are physically linked and can only bind in one orientation relative to each other. For cooperatively acting transcription factors though, there could be variation in the orientation of the sites relative to each other. To account for this, we can adapt the gap surprisal function to:
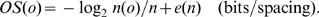
(3)where we calculate an orientation surprisal (*OS*(*o*)) that is the logarithm of the frequency of occurrence at each orientation. For a system where both orientations occur at equal frequency, the number of occurrences at either orientation would be *n*(*o*) = 1, and the total number of occurrences is *n* = 2. The orientation surprisal for this system would therefore be 1 bit of information. In a system where there is no variability in orientation, the frequency of occurrence at that orientation would be *n*(*o*)/*n* = 1, and therefore the orientation surprisal would be 0 bits. The advantage of the *OS*(*o*) calculation is that we can model the subtle energetic differences for systems that allow either orientation, but favor one over the other.

To calculate the total information for Met4 coordination, we can now expand equation (1) to: 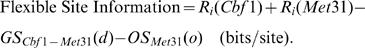
(4)There is no orientation surprisal for Cbf1. Since Cbf1 is homodimeric and has a symmetric matrix, the Cbf1-DNA complex would be identical for either orientation. In this case, the frequency of occurrence of a given orientation would be 1, and *OS_Cbf_*_1_ = 0 bits. Therefore, the orientation surprisal only applies to asymmetric binders.

Combinatorial scans were done using **multiscan** [10] to identify and quantify Cbf1/Met31 cooperatively acting binding sites in the genome. The individual information contribution for both sites (*R_i_*(*Cbf*1) and *R_i_*(*Met*31)) were calculated over the range −4 to +5, since this is the range of conservation for both logos (Fig. 1) [20]. Sites were only considered if each component had an *R_i_*>0 bits (which would correspond to a -Δ*G* of binding [20], [25]) and they have a flexible site information >0 bits. For a site to have a positive flexible site information, the ordering and orientation of the pair have to be within the defined spacing and ordering parameters. For any spacing or orientation outside of the specified range, the sites would have a surprisal penalty equal to infinity according to equations (2) and (3), and a flexible site information <0 bits according to equation (4).

All genes in the genome were then ranked based on the strength of their strongest upstream site. Microarray expression data for sulfur amino acid pathway-affected cells (see Microarray Datasets) were then averaged for the top 30 genes in our ranking. All values averaged were log_2_ of the expression fold change between affected and unaffected cells. This was done independently for induction and repression experiments.

The physical constraints that we want to define are: the ordering of the sites relative to the gene start, the orientation of the matrices, the maximum allowed distance between Met4 and the polymerase binding site, and the spacing range between Cbf1 and Met31 that can bind Met4. We varied these constraints, and iteratively refined the model to get the optimal predictor. We evaluated any given set of parameters by calculating the average expression change in the top 30 ranked genes. The greater the expression change the better the model.

Another approach could be to cluster genes based on similar trends in expression data across several experiments, and then try to train our parameters based on this set of genes. One disadvantage of this is that it is difficult to discern directly from indirectly regulated genes in these clusters. By scanning the genome and ranking the genes, we are selecting only for genes that are directly regulated. Also this approach does not exclude genes that are regulated but had anomalous expression data due to experimental error. Since there have been at least 20 genes implicated in sulfur assimilation [5], we chose to average the top 30 gene expression differences to evaluate our model. We chose 30 so that we would not overfit our model by looking at too few genes, and not introduce noise into our analysis by averaging too many.

### Microarray Datasets

We used microarray data from two sources for our analysis. Gasch *et al.* [26] reported amino acid starvation data, where transcription of Met4 regulated genes was induced. Fauchon *et al.* [27] reported Cd^2+^ addition experiments where Met4 regulated genes were induced, and Met4 deletion experiments where Met4 regulated genes were repressed. Our models were optimized against these data as mentioned above. Microarray expression patterns were visualized using **TreeView** [28]. The yeast genome sequence and annotation that we used in our analysis came from Genbank accession numbers NC001133 to NC001148.

## Results

### Cbf1 and Met31 logos

Since Cbf1 is a homodimeric protein, we used all sequences and their complements to build our model [21]. Conservation at positions −2,−1 and +2,+3 is strong and does not match the helical accessibility wave (Fig. 1A). Deviation of sequence conservation from the helical accessibility wave is generally an indicator of structural changes in the DNA substrate [29]. This may be consistent with the observed bending of DNA by Cbf1 [30].

The Met31 model was built as described in Materials and Methods (Fig. 1B). Sequence conservation appeared to follow the helical accessibility wave well, and it was contained within one major groove. Met31 has an asymmetric binding site, so it can possibly bind with two different orientations. We tested both orientations in our analysis. The information content for the Cbf1 logo is 12.9 bits over the range −4 to +5. The information content for the Met31 logo is 11.9 bits over the range −4 to +5.

### Orientation and ordering

**Figure 2:**
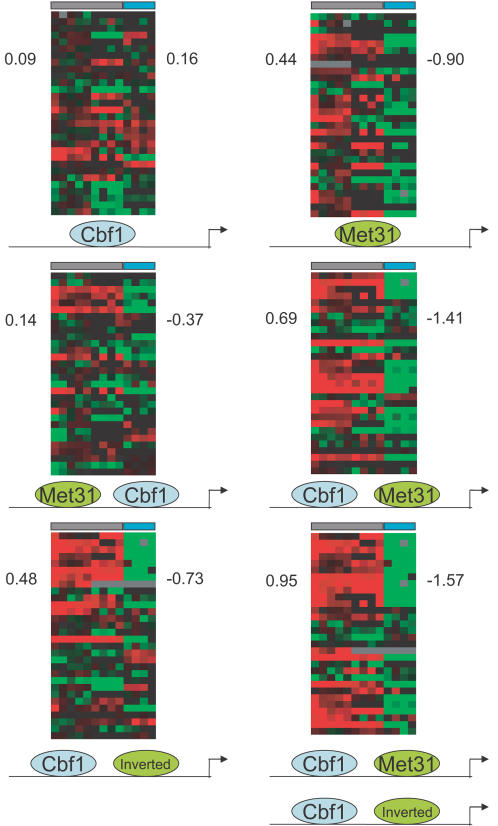
Met4 binding by Cbf1 and Met31 is dependent upon ordering but not orientation. We scanned all intergenic regions in yeast with the models presented in Fig. 1 with different orientations and orderings relative to the gene start point. We then ranked all genes in the genome based on the strength of their strongest upstream binding site, and we present here the corresponding expression changes as determined by microarrays. The experiments that each column represent correspond to those in Fig. 6. For columns 1–9 (marked with a gray box) we expect regulated genes to have increased expression and therefore to be red. For columns 10–13 (marked with a blue box) we expect regulated genes to have a decreased expression and therefore to be green. Since the Met31 matrix is asymmetric, it could bind with two different orientations. Those circles labeled “Met31” have the same orientation as the Met31 logo in Fig. 1. Those circles labeled “Inverted” have the opposite orientation (see Fig. 5). The optimal combination in the lower right corner allows for either orientation of Met31. The arrow signifies the gene start. The average expression change for the top 30 genes was calculated for each combination of sites for both the induced (columns 1 to 9) and repressed (column 10 to 13) experiments and are reported next to their respective columns.

Since Cbf1 and Met31 are not physically linked upon binding, it was not immediately obvious what the ordering and orientation constraints on their binding are in functional Met4 docking complexes. To determine this, we tested the predictive capabilities of all combinations of orientation and ordering for Cbf1 and Met31 using the gene-ranking approach described in Materials and Methods. Briefly, we determined the flexible information for the cooperative model as determined by equation (4) [9], [10], and ranked all genes in the genome based on the strength of the strongest site in the intergenic region immediately upstream of their starts. We then calculated the average expression fold change of the top 30 genes in this ranking based on Met4 induced and repressed microarray experiments [26], [27]. We regarded those combinations that gave the highest average microarray expression change to be the optimal organization for Met4 coordination. Fig. 2 shows how well different combinations performed.

Cbf1 alone was not sufficient to identify the Met4 regulated genes. The average expression fold change for the top 30 ranked genes was 0.09 and 0.16 for induction and repression data respectively, we report corresponding values for all other combinations. Met31 alone appeared to be a better predictor than Cbf1, but was still weak (0.44 and −0.90). This improvement of Met31 prediction over Cbf1 is expected since Cbf1 has a regulatory role outside of Met4 binding [31]. For both Cbf1 and Met31, we only considered binding sites within 1000 bases upstream of the closest gene start. By searching for Cbf1 and Met31 sites together, with a maximum spacing of 100 bases between the zero positions of the binding components (Fig. 1) and the downstream component could be a maximum of 1000 bases upstream of the gene start, the prediction was better. If we searched with the order Cbf1-Met31-gene start, we were able to identify more genes with the expected microarray pattern than with the order Met31-Cbf1-gene start (0.69 and −1.41 vs. 0.14 and −0.37).

Since Cbf1 is a homodimer, its binding is independent of orientation. Since Met31 is monomeric, its binding is orientation dependent. When we allowed for both orientations of Met31 downstream of Cbf1, we got the largest change of expression (0.95 and −1.57). This suggested that transcriptional activation by Met4 requires a Met31 site with any orientation to fall between Cbf1 and the gene start (bottom right panel of Fig. 2).

To test whether the average expression values that we observed are statistically significant, we randomly chose 10,000 sets of 30 genes from the genome and averaged their expression change values. We did this for both the induced and repressed data sets. Both sets gave similar normal distributions with a mean of −0.015 and SD of 0.11 for the induced data set and a mean of 0.001 and SD of 0.094 for the repressed data set. For the best organization of sites in Figure 2, an expression change of 0.95 and −1.57 would be 8.7 and 16.7 standard deviations from the mean respectively. The probability of selecting a set of 30 genes with an average expression change this high randomly would be less than 1×10^−8^.

All models for the remainder of this analysis will have these ordering and orientation requirements imposed on them. The designation of the Met31 model orientation as “normal” or “inverted” is arbitrary. We also tested the “inverted” Met31 model alone, and inverted Met31 upstream of Cbf1, but the results were similar to equivalent scans with the “normal” orientation (data not shown).

### Spacing constraints

There are two spacing constraints on this system, the distance between the Met4 docking complex and the initiating polymerase, and the distance between the two binding components (Cbf1 and Met31) within the Met4 docking complex. To define what these spacing ranges are for functional Met4 binding sites, we systematically modeled different spacing ranges, and quantified the models by the gene-ranking approach previously described. Interestingly, if we varied one of the spacing constraints, the optimal spacing for the other would differ slightly. To identify which spacing parameters define the optimal predictor, we varied both spacings simultaneously, and quantified their predictability by averaging the expression change of their 30 highest ranking genes.

We increased the maximum allowed distance of the Met4 docking complex from the gene start in 50 base increments as measured by the distance between the Met31 site and the translational initiation codon. At each 50 bp increment, we varied the minimum and maximum allowed distance between Cbf1 and Met31 from 1 to 100 bases. These distances are relative to the zero position of both matrices (Fig. 1). We then summed the average expression change for the induction and repression experiments for all combinations of spacings, and determined which combination predicted the microarray data best.

**Figure 3:**
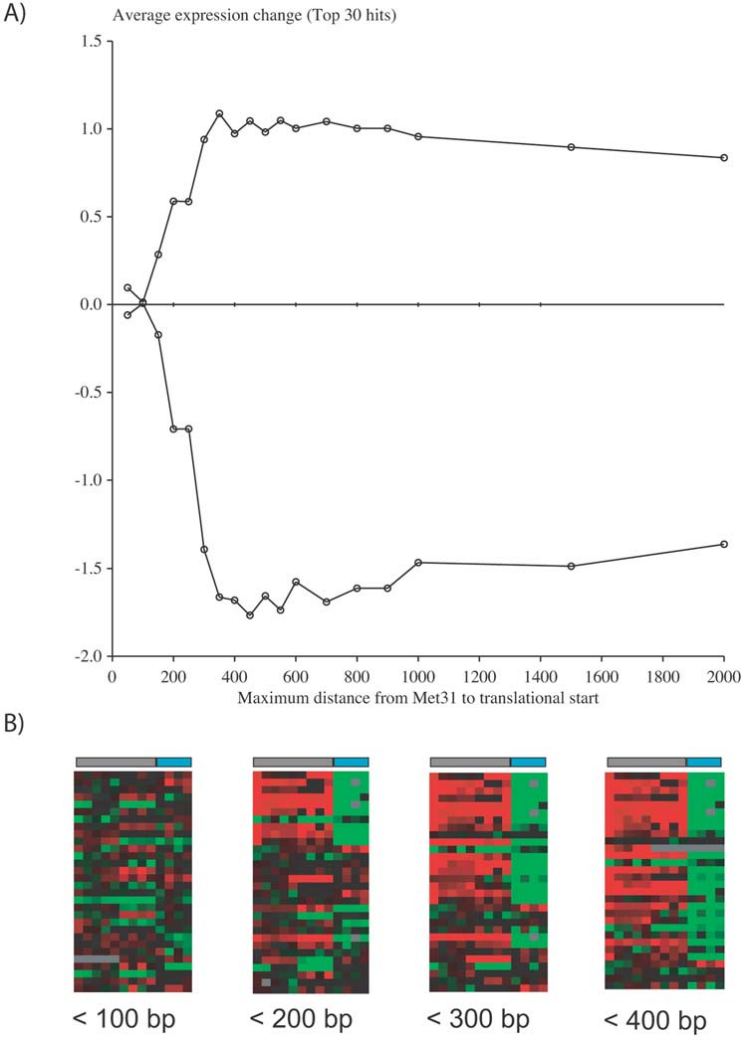
Met4 binding is within 450 bases of the gene start, but not within 100 bases. We varied the allowed distance that the Met31 binding site can be from the gene start point in our models, and quantified how this spacing constraint affected our ability to predict microarray expression data. A) We plotted the average expression change of the top 30 hits in the genome for different maximum spacings from the gene start. The top line corresponds to data from experiments where we expected increased expression (columns 1 to 9 in B), and the lower line is from experiments where we expected decreased expression (columns 10 to 13 in B). The microarray data that corresponds to our gene-ranking are shown in B. The conditions for each column in the microarrays correspond to the labeled columns in Fig. 6.

For the first spacing constraint, the distance between Met4 and the polymerase, we found the optimal maximum spacing was 450 bases (Fig. 3). The predictability of the model seemed to increase linearly from 100 to 350 bases suggesting that the sites are evenly distributed over this range. There appeared to be few or no genes with sites closer than 100 bases upstream, or sites farther than 450 bases upstream that had the expected expression pattern.

**Figure 4:**
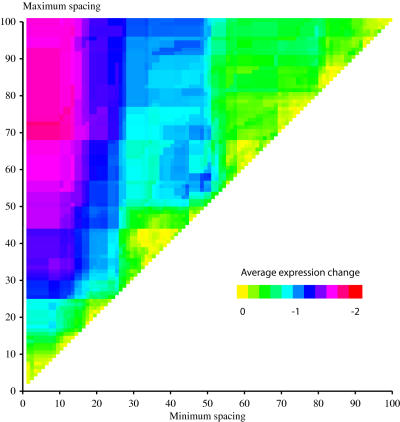
The optimal spacing range between Cbf1 and Met31 is 9 to 68 bases. We varied the minimum (X axis) and maximum (Y axis) distance that Cbf1 and Met31 could be from each other in our model, and calculated the average expression change within the corresponding top 30 hits, according to these ranges. We show here the average expression change for only those experiments that we expected to have a decreased expression (columns 10 to 13 in Fig. 6). The colors correspond to the key in lower portion of the plot.

For the second spacing constraint, the distance between Cbf1 and Met31, we found the optimal spacing range to be −9 to −68 bases, the minimum to maximum spacing allowed between each site (Fig. 4). This was the range used in the analysis in Fig. 3. Ranges close to −9 to −68 appeared to have a similar level of predictability as indicated by the redish semi-circle in Fig. 4, but −9 to −68 had the highest expression change and the tightest range. The average expression changes for these two spacing parameters were 1.04 and −1.77 for induction and repression data respectively.

### Optimal model

**Figure 5:**
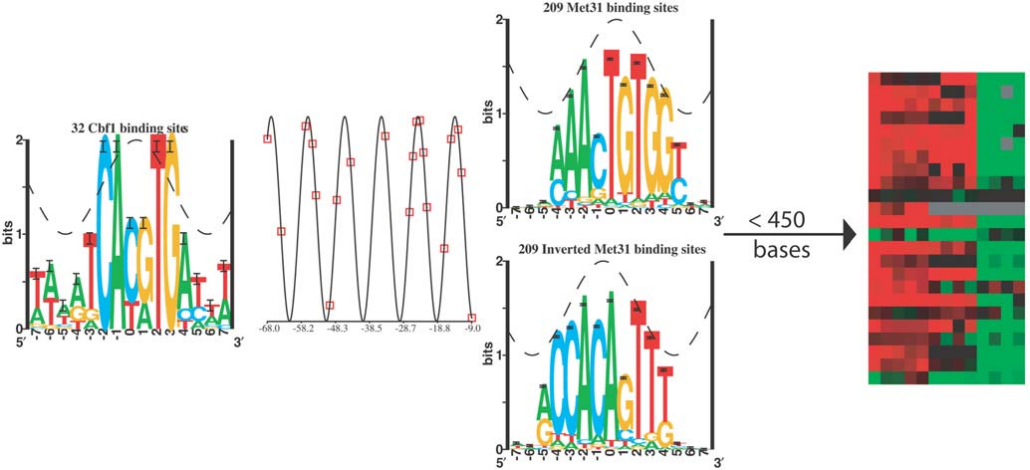
The Met4 activation model based on our analysis. We summarize here the spacing, ordering, and orientation constraints we used to define functional Met4 binding sites. Since Met31 can bind with either orientation, we show logos for both Met31 orientations. The distances between each set of Cbf1 and Met31 sites were plotted with red boxes on a cosine wave for 23 high-ranking genes to show helical preferences. The arrow represents the translational start, and the allowed distance between the Met4 stabilization complex and the translational start is written above it. The expression data on the right is what was predicted by this model, and is described in Fig. 6.

**Figure 6:**
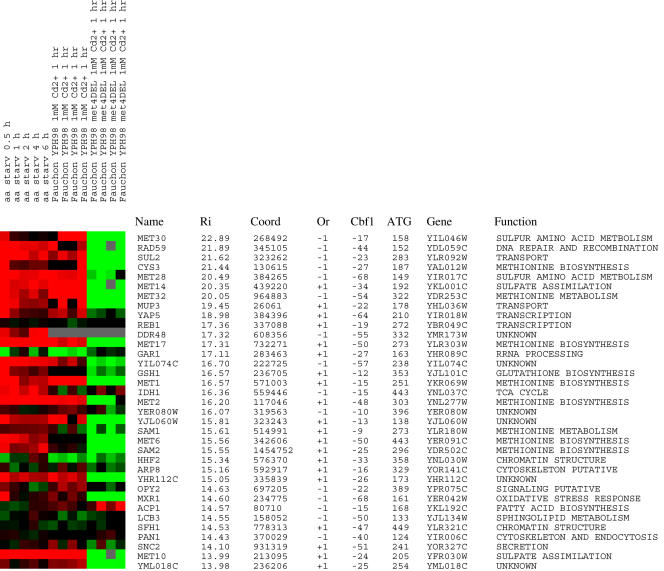
The top hits are involved in sulfur amino acid biosynthesis. These are the top hits according to our optimal spacing values. The first 9 columns are data for experiments that should induce the expression of Met4 regulated genes and give a red pattern. The last 4 columns we expect to see a decrease in expression of Met4 regulated genes and give a green pattern. Experiment information for each column is reported vertically above each column. Each row corresponds to a different gene followed by its common name, its flexible information (*R_i_*), the coordinate of the Met31 binding site in the *S. cerevisiae* genome, the orientation of the Met31 matrix, the distance Cbf1 is upstream of Met31, the distance the gene start is downstream of Met31, the gene name according to its annotation in the yeast genome, and a description of its function.

Based on the analysis in Fig. 2, Fig. 3, and Fig. 4, the optimal model is shown in Fig. 5. This model requires a Cbf1 site to be 9 to 68 bases upstream of a Met31 site with either orientation, and for the Met31 site to be no more than 450 bases upstream of the translational initiation codon. When we scanned the genome with this model, we see that most of our top hits are genes known to be involved in sulfur amino acid biosynthesis (Fig. 6). Two genes in the top 23 hits have a strikingly unexpected expression pattern (Reb1 and Gar1). Additional analysis of these sites show that they both have a strong Cbf1 site, but a “T” instead of “G” at position +1 of their Met31 site. This suggests that the information contribution at position +1 may be greater than that in our current matrix. Several genes have both the expected expression profile and a predicted Met4 binding site, but their functions have not been biochemically characterized (DDR48, YIL074C, YJL060W, YHR112C). Clustering of co-regulated genes by the gene-ranking method may have identified other genes involved in sulfur utilization.

To test whether we were over- or under-fitting our model, we repeated the optimization by averaging the top 20 and 40 genes instead of 30. When we did this our results differed slightly. By averaging the top 20 genes, we found the optimal spacing range shrunk slightly to 13 to 68 bases between Cbf1 and Met31 and the optimal maximum distance between Met31 and the gene start remained at 450 bases. At this smaller range, Gsh1 and Sam1 were lost, both which have been implicated in sulfur assimilation. By averaging the top 40 genes, the spacing range remained the same (9 to 68 bases), but the maximum spacing range expanded to 750 bases. No new genes with the expected expression pattern or functional evidence for Met4 regulation were identified at this larger spacing. The larger the number of genes that are averaged, the more likely random genes with above average expression differences will be in the averaged set, which will obscure the actual parameters. Thirty genes appeared to be the appropriate number to average, and the relatively consistent parameters observed from averaging 20, 30, or 40 genes suggested that these parameters are reliable.

To test whether there is a tendency for Cbf1 and Met31 to bind on the same face of the DNA, we plotted the relative spacing between the two sites on a cosine wave with the same period as B-form DNA, 10.6 bases (Fig. 5). We plotted the spacings of 19 of the 23 top ranking genes (all sites except for Reb1, Gar1, Idh1 and YER080W) and YHR112C, Mxr1, Met10, and YML018C which had both a strong flexible information and expression change. To determine what the optimal phase of the cosine wave was, we plotted each spacing on a cosine wave and calculated the average height of all spacings on the helix. That is, if all spacings were at the top of the cosine wave (occurred in multiples of 10.6 bases) then the average helical location would be high. We determined the phase of the cosine wave that gave the highest average helical location of these 23 spacings, and found the optimal phase to peak at −13.86 bases relative to the Met31 zero position. To see if the relative placement of these spacings on the cosine wave is higher than expected, we determined the average helical location of random sets of 23 Cbf1/Met31 pairs. Our set had an average helical positioning greater than 95 percent of random sets.

Sam1 has the shortest Cbf1-Met31 spacing of 9 bases. This spacing is completely on the opposite face of the DNA according to a phasing of −13.86 bases. One would expect spacing to be more constrained at shorter distances, since it is more difficult for DNA to bend. We looked at the region upstream of the Sam1 translational start to see if there was another strong Cbf1-Met31 pair with a larger spacing. We found a second weaker Cbf1 site 36 bases upstream of the Met31 site, giving a total flexible information of 12.6 bits. This spacing would suggest same face binding according to a phasing of −13.86 bases. The strong Cbf1 site 9 bases upstream of the Met31 site may have occurred randomly, or may be part of an overlapping second site.

To calculate the flexible individual information for each binding site, we used equation (4). Since we did not know the energetic effect of different spacings on the complex initially, we treated all spacings equally. That is, over the range 9 to 68 (60 bases of variability) all positions had the same gap surprisal of *GS*(*d*) = -*log*_2_(1/60) = 5.91 bits according to equation (2). We also assumed an equiprobable occurrence of each orientation of Met31, so that *OS_Met_*_31_(*o*) = -log_2_(1/2) = 1 bit of information according to equation (3). Therefore the *GS* and *OS* variables in equation (4) effectively become constants summing to 6.91 bits of uncertainty for each site. Because of the small number of target genes, and the already strong predictive capabilities of our model, we cannot determine the individual spacing constraints for this system. If we had a system with more sites, robust spacing and orientation distributions could be determined and individual penalties could be assigned.

We can use these values to predict the *R_sequence_* or average information content for this system which is:
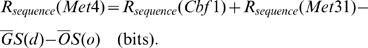
(5)


*G̅S*(*d*) is the mean *GS*(*d*) value for all sites, and *ŌS*(*o*) is the mean *OS*(*o*) value. According to this equation *R_sequence_*(*Met*4) = 12.9+11.9−5.91−1.0 = 17.9 bits of information.

**Figure 7:**
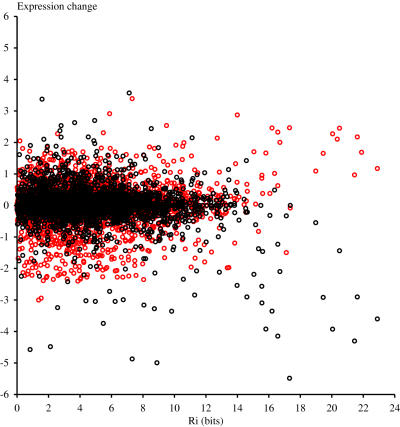
R_i_ vs. expression change. The flexible information (*R_i_*) of the strongest site for each gene is on the abscissa and the average induction or repression expression change is on the ordinate. For each gene, induction data were averaged from the first nine experiments in Fig. 6 (red circles), and repression data were averaged from the last four experiments in Fig. 6 (black circles).

For each gene we plotted the strength of its strongest upstream Met4 binding site according to the model in Fig. 5 and its average expression change for induction and repression experiments (Fig. 7). At about *R_i_*>14 bits, the number of genes that showed no, or an unexpected expression difference was significantly lower. This is about the same *R_i_* as the site upstream of Met10 (14.0 bits), the lowest ranking sulfur assimilation protein in our analysis.

## Discussion

Transcriptional initiation in eukaryotes is often regulated by multiple cooperatively acting factors. Often these factors can only positively affect transcription if they physically interact either directly or indirectly through additional proteins with the basal transcriptional machinery. Understanding the physical constraints that determine functional cooperativity is essential for us to be able to model, predict, and engineer genetic control systems. These constraints generally are not rigid, but allow for variability in the arrangement of sites in functional complexes and subsequently there is variability in the stability of the complexes. Here, we have introduced a way to include orientation and order into the information theoretic description of pattern recognition at the promoter. This combined with weight matrix based binding models [20] and spacing constraints [9], [10] gives us quantitative tools to model the sequence basis of eukaryotic transcriptional regulation.

The simplest constraint of Met4 coordination to define is the ordering of the sites within the complex. For Met4, our model matches microarray data poorly when the order is Met31-Cbf1-gene start, but matches well with the order Cbf1-Met31-gene start (Fig. 2). This is consistent with experimentally determined ordering constraints [32]. These results suggest that the Met4-Cbf1 binding surface is distinct from the Met4-Met31 surface, and that the Met4-TFIID binding surface is closer to the Met4-Met31 surface, placing the Met4-TFIID binding surface near the 3′ edge of the complex. Domain mapping from yeast two-hybrid experiments identified several protein interaction domains on Met4 [4]. The transcriptional activation domain (residues 95–144) is closer to the Met31 interaction domain (residues 374–403) than the Cbf1/Met28 interaction domain (residues 616–666) in one-dimension, but these domains are far apart, so their relative positioning in the native form of Met4 could be different. Based on our findings, we suggest that the relative positioning is the same.

It appears as though either orientation of Met31 can be used within the docking complex (Fig. 2). Stabilization of Met4 has been shown when Met31 has the inverted orientation [4]. For Met31 to be able to stabilize Met4 with either orientation, it must either have two Met4 interaction surfaces, or it has a centrally located interaction surface that is accessible no matter what orientation it binds (*i.e.* flexible). Interestingly, the top 5 genes in our ranking have an inverted Met31 site (Fig. 6). The total information of these sites might be high because stronger Cbf1 and Met31 sites may be necessary to compensate for the strain of the inverted orientation, but could decrease once we take into account the orientation surprisal.

The maximum distance between the Met4 docking site, as measured to the zero coordinate of the Met31 site, and the gene start is 450 bases. As the TFIID binding site is not at the gene start, this distance is farther than the maximum allowed distance between Met4 and the polymerase. It is difficult to determine the distance of the Met4 docking complex to the transcriptional start since the starts have not been biochemically proven, and computationally it is difficult to predict transcription initiation because of the varied modes of initiation by the polymerase [2]. Basehoar *et al.* found an enrichment of TATAs between 50 and 200 bases upstream of the translational start [33]. This could explain why we did not observe any sites within 100 bases of the gene start.

The spacing range between the Cbf1 and Met31 site is 9 to 68 bases, as determined in Fig. 4. The minimum spacing of 9 bases may be an under-estimate. There is a strong secondary Cbf1 site 36 bases upstream of the Met31 site in the promoter of Sam1, which may be the actual functioning site. A similar strong secondary site is also seen for YER080W, which had a spacing of 10 bases. This is not seen for GSH1, which had a spacing of 12 bases.

A minimum spacing of 12 would be consistent with our observed optimal helical phasing of -13.86 bases (Fig. 5). This would place the closest Cbf1 site almost exactly on the same face as its respective Met31 site, one helical turn away. A maximum spacing of 68 bases would correspond to 6 helical turns according to our phasing. The relatively high positioning of these spacings on the helical accessibility curve suggests that docking of Met4 may be dependent upon the helical phasing of DNA.

The experimentally determined range by Chiang *et al.* was 21 to 53 bases according to our numbering system [32]. Unfortunately, spacings as large as 68 bases were not tested experimentally. The experimentally determined minimum spacing of 21 is much larger than the minimum we found here. Interestingly, only the “inverted” orientation of Met31 was tested, whereas the shortest distance in this paper corresponds to a Met31 site with the opposite orientation. If helical phasing of the sites is important, then the orientation of Met31 may be more constrained at shorter distances, and this may account for the disparity between the experimentally and computationally determined minimums.

When the constraints inferred from our analysis were imposed on the cooperative binding of Cbf1 and Met31, our ability to predict Met4 regulation was high. Of the top 23 ranked genes in *S. cerevisiae* (according to our model), 19 had an expected microarray expression pattern for Met4 regulation. Many of the sites had also been previously characterized as sulfur utilization genes (Fig. 6). The 2 most striking anomalous genes in the top 23 (Reb1 and Gar1) both had Met31 sites with a “T” instead of “G” at position +1 (data not shown), suggesting that this position may be weighted more strongly in a more refined Met31 model. Additionally, nucleosomes could play a large inhibitory role against spurious combinations of sites, which our model does not account for.

When the microarray data from experiments that affected Met4 binding were directly compared to our information evaluation of each gene (Fig. 7), we saw that almost all genes with Met4 binding sites above 14 bits of information have the expected expression change. This suggests that our approach is giving some reasonable estimate of the energetics of Met4 binding, with a clearly defined threshold for functional binding sites. Presumably, genes that do not have a strong Met4 site, but have the expected microarray data are indirectly regulated. Interestingly the strengths of the Met4 sites are not mainly determined by the strength of Met31 or Cbf1, but by the sum of these sites. This suggests that for cooperatively acting binding sites, a decrease in strength for one site can be compensated for by an increase in strength of the other. Compensation for a decrease in the strength of one binding site by increasing the affinity for another site has been shown experimentally for activation of Pol II by the Epstein-Barr virus protein ZEBRA [34].

We cannot determine the individual spacing effect on binding because we have so few sites covering a large spacing range. If we did have these individual effects, we would expect to see a slight improvement in our ability to predict expression. The fact that we did so well in predicting Met4 sites by just taking into account the strength of the sites is surprising to us. In bacteria, transcription factors interact at shorter distances, and the effect of spacing on stability is greater (since it is more difficult to bend a short piece of DNA than a large one) [10]. For coordinated binding of Met4, the summed affinities of Cbf1 and Met31 appeared to dominate the stability of the Met4 complex, suggesting that the energetic penalties associated with spacing are considerably less for this system. Experimental testing of multiple spacings between Cbf1 and Met31 suggested that spacing had a little effect on Met4 regulation [32]. A decrease in the effect of spacing on stability could be due to increasing the flexibility between the activation domain and the DNA-binding domain of the transcription factors, by increasing the distance between functioning cooperatively acting binding sites, or by increasing flexibility in the coordinating protein.

We could have determined these physical constraints by clustering co-regulated genes and training the rules of binding for their regulators. The drawback of this approach is that it is not obvious which genes are directly and indirectly regulated, and a given gene may or may not have a binding site. Our approach selects only for genes that are directly regulated, and does not exclude sites that have poor expression data due to experimental error. We are also optimizing our model against all genes in the genome, so we are selecting for a model that represents Met4 binding well, in that it can identify a small subset of sites from all sites in the genome. Presumably the optimal binding site, based on the flexible information theory approach, is the most stable site and the easiest to crystalize. These results could be used to guide crystallographic experiments.

The information content of a given DNA-binding protein (*R_sequence_*) is a function of the variability within its binding targets [24]. A more stringent binder would have a higher information content, since the variability in its binding targets would be smaller. To be able to distinguish **γ** binding sites within a random DNA of some length **G**, those sites must have an *R_frequency_* = -*log*_2_(γ/*G*) bits of information to be identified [24]. It has been shown for many systems that *R_sequence_* converges to *R_frequency_* [24], [35]. This suggests that if the size of the genome increases and the number of binding sites remains constant, the information of those sites would have to increase in order to be distinguishable.

As eukaryotic genomes are generally larger than prokaryotic genomes, the amount of information needed to identify **γ** sites would have to be greater. This can be achieved either by increasing the information content of a single factor, or by using multiple factors combinatorially.

Assuming no individual spacing or orientation preferences, the information for this system would be 12.9+11.9−5.9−1 = 17.9 bits according to equation (4). This would correspond to 1 site every 2^17.9^ = 2.45×10^5^ bases, or about 104 times in the *S. cerevisiae* genome of length 12.8 MB. Our calculation is the number of sites in 2× the genome length, since the complex could associate with either strand. This is a reasonable number of genes according to known sulfur assimilation genes (>20 genes) [5], the number of predicted regulated genes based on expression difference due to Cd^2+^ treatment (66 genes) [27], and multiple sites per gene as seen in several cases. This suggests that like single acting transcription factors, the information contained in combinatorial binders is related to the *R_frequency_* for that system [24]. Others have suggested that this relationship will be maintained for cooperatively acting factors [36]. Interestingly, this information is distributed through individual binding components, as well as the spacing between components, and if one component changed, the others would have to compensate accordingly. This is a complicated process, since Cbf1 can also function independently of Met4 and Met31 [31].
